# Electrolysis for the Removal of Superfluous Hairs

**Published:** 1902-05-03

**Authors:** 


### ELECTROLYSIS FOR THE REMOVAL OF SUPERFLUOUS HAIRS.

It is much to be regretted that so many medical practitioners are so unenterprising, or perhaps are so tied down to their accustomed "bottles of physic," that new methods of treatment, however successful they may be, if they do but require a little special apparatus or the acquisition of a little knack, are so often allowed to slip away into the hands of unqualified persons. Take for example the case of electrolysis for the removal of superfluous hairs. No one can doubt that this is truly a surgical operation, yet how largely it has passed into the hands of unqualified persons! Dr. David Walsh,[1] writing in regard to this process, says that the only really efficient method of removing superfluous hair from the face is by electrolysis. Depilatories merely palliate the evil for the time being, while in the long run they cause the growth of the hairs to be redoubled. The process is tedious and painful, but these obstacles are surmountable with a little determination on the part of the patient. Much harm is done to the credit of the operation by claiming too much, saying, for instance, "that the pain is trifling, that the destruction of the hair is certain, and that no mark is left," for it may be affirmed that "it is impossible to remove deep-coarse hair without a fair amount of pain, and that some slight scarring must necessarily result." As to recurrence, it is admitted by most medical writers that re-growth of the hair takes place in a fair percentage of the follicles operated upon. This is due to the varying and sometimes twisted direction of the follicle, but a second electrolysis of the recurring hair is likely to end in the complete destruction of the bulb. Finally, Dr. Walsh urges that the performance of this little operation should be kept in the hands of the profession.

[1] Lancet, April 12.

